# Supplementary material for: Peptidomimetic inhibitors of TMPRSS2 block SARS-CoV-2 infection in cell culture
Source: Commun Biol. 2022 Jul 8;5:681. doi: 10.1038/s42003-022-03613-4 (PMC9270327; doi:10.1038/s42003-022-03613-4)
Supplement: Supplementary file 2 — Supplementary Information [file 42003_2022_3613_MOESM2_ESM.pdf]

Supporting Information

for the article

Peptidomimetic Inhibitors of TMPRSS2 block SARS-CoV-2 Infection in Cell Culture

Lukas Wettstein[1#], Philip Maximilian Knaff[2,3#], Christian Kersten[4#], Patrick Müller[4], Tatjana Weil[1], Carina Conzelmann[1], Janis Müller[1], Maximilian Brückner[2,3], Markus Hoffmann[5,6], Stefan Pöhlmann[5], Tanja Schirmeister[4], Katharina Landfester[3], Jan Münch[1,7*], Volker Mailänder[2,3*]

[1] Institute of Molecular Virology, Ulm University Medical Center, 89081 Ulm, Germany

[2] Dermatology Clinic of the University Medicine of the Johannes Gutenberg University Mainz, 55131 Mainz, Germany

[3] Max Planck Institute for Polymer Research, 55128 Mainz, Germany

[4] Institute of Pharmaceutical and Biomedical Sciences, Johannes Gutenberg University Mainz, 55128, Mainz, Germany

[5] Infection Biology Unit, German Primate Center, 37077 Göttingen, Germany and

[6] Faculty of Biology and Psychology, Georg-August-University Göttingen, 37073 Göttingen, Germany

[7] Core Facility Functional Peptidomics, Ulm University Medical Center, 89081 Ulm, Germany

[#] These authors contributed equally.

[*] These authors jointly supervised this work.

Corresponding authors: jan.muench@uni-ulm.de; mailaend@mpip-mainz.mpg.de

Supporting Figures:

Supplementary Figure 1 Analytical data (structure, HPLC, MS, NMR data) of precursors and serine traps, p.3-9.
Supplementary Figure 2 Michaelis-Menten constant (K_M) of the fluorogenic reference substrate Boc-Gln-Ala-Arg-AMC for TMPRSS2, p.10.
Supplementary Figure 3 Validation of molecular docking models, p.11.
Supplementary Figure 4 Analytical data (structure, HPLC, MS) of peptidomimetic inhibitors, p.12-14.
Supplementary Figure 5 Superposition of TMPRSS2 models and crystal structure, p.15
Supplementary Figure 6 Influence of serine trap on biological activity of compound 7 against matriptase, thrombin and factor Xa, p.16.
Supplementary Figure 7 The transmembrane serine protease TMPRSS2 is expressed on Caco-2 cells, p17.
Supplementary Figure 81 Impact of DMSO on SARS-CoV-2 spike mediated entry and infection, p.18.
Supplementary Figure 9 Cytotoxicity of DMSO, p.19.
Supplementary Figure 10 UV absorbance profile (220 nm) of RP-HPLC chromatograms of 7 incubation with human serum, p.20.
Supplementary Figure 11 Influence of (*S*)- and (*R*)- epimers of compound 2 (ace-Arg-Pro-Arg-kbt) on inhibitory activity against matriptase, p.21.
Supplementary Figure 12 Chromatogram of (*S*)- and (*R*)- epimer of compound 2, p.22.
Supplementary Figure 13 Plasma stability of inhibitors, p.23.
Supplementary Figure 14 Cell culture medium stability of inhibitors, p.24.

Supporting Tables:

Supplementary Table 1 FlexX-scores of tripeptidic substrate-analogue ligands for docking receptor validation, p.25.
Supplementary Table 2 Docking results of P1-sidechain screening of peptidomimetic inhibitors with ace-Pro-X-aldehyde sequence for 7 potential basic amino acid-derivatives, p.25.
Supplementary Table 3 Excerpt of docking results of P2-sidechain screening of peptidomimetic inhibitors with ace-D-Arg-X-Arg-aldehyde sequence, p.25.
Supplementary Table 4 Excerpt of docking results of P3-sidechain screening of peptidomimetic inhibitors with ace-X-Pro/Gly-Arg-aldehyde sequence, p.26.
Supplementary Table 5 Influence of serine trap on biological activity of compound 7 against matriptase, thrombin and factor Xa, p.26.
Supplementary Table 6 IC_{50} values of peptidomimetic TMPRSS2 inhibitors, camostat mesylate (CM) and FOY-251 measured on Caco-2 or TMPRSS2 expressing HEK 293T cells, p.26
Supplementary Table 7 IC_{50} values of peptidomimetic TMPRSS2 inhibitors and camostat mesylate (CM) against SARS-CoV-2 spike -pseudotyped lentivirus, p.27.
Supplementary Table 8 IC_{50} values of peptidomimetic TMPRSS2 inhibitors and camostat mesylate (CM) against SARS-CoV-2 Wuhan-Hu-1 and variants of concern, p.27.

i) **Boc-Arg(Mtr) Weinreb amide (S1)**

((S)-tert-Butyl-(1-(methoxy(methyl)amino)-5-(3-((4-methoxy-2,3,6-trimethylphenyl)sulfonyl)guanidino)-1-oxopentan-2-yl)carbamate)

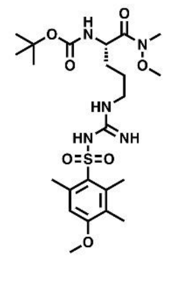

529.26 g/mol

HPLC

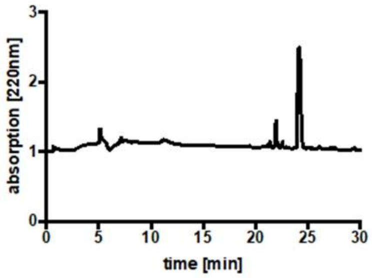

Retention time 24 min.
Purity (HPLC, 220 nm) > 80%.

NMR (300 MhZ, CD3OD)

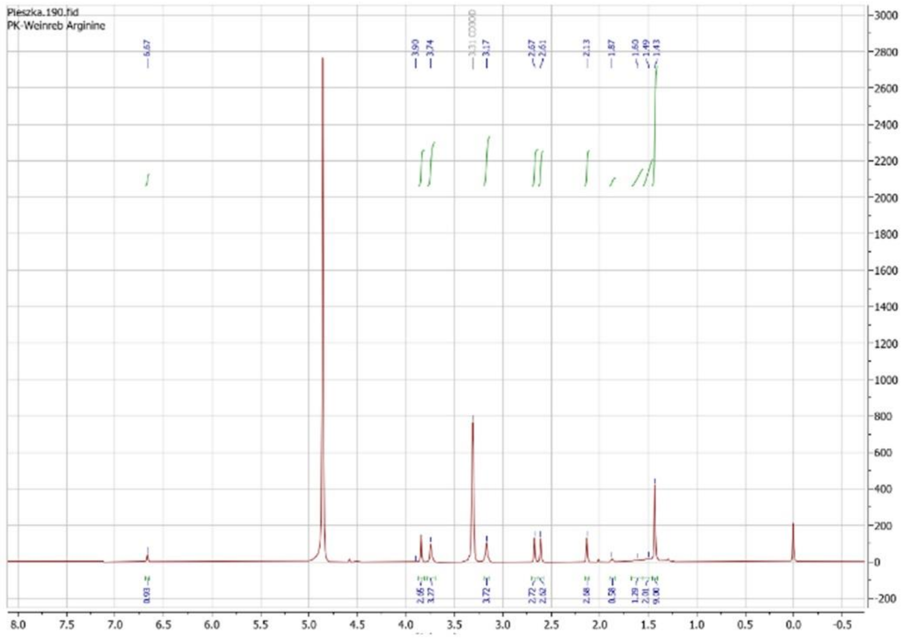

^{1}H NMR (300 MHz, CD3OD): δ=1.43 (s, 9 H) 1.49–1.60 (m, 4 H) 1.87 (s, 1 H) 2.13 (s, 3 H) 2.61 (s, 3 H) 2.67 (s, 3 H) 3.17 (m, 3 H) 3.74 (s, 3 H) 3.90 (s, 3 H) 6.67 ppm (s, 1 H).

MS-ESI

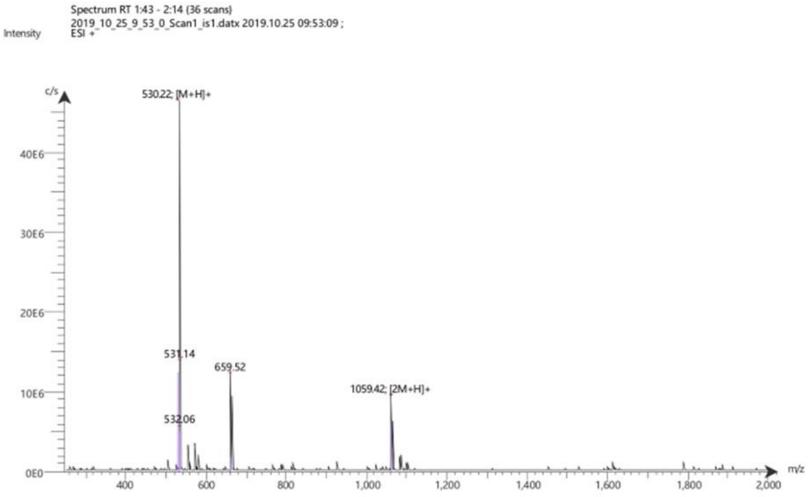

ESI (MS) *m/z*: calcd for $C_{23}H_{39}N_5O_7S_2$ $[M+H]^+$ 530.3, $[2M+H]^+$ 1059.6, found $[M+H]^+$ 530.2, $[2M+H]^+$ 1059.4

ii) **Boc-Arg(Mtr) ketobenzothiazole (S2)**

tert-Butyl-(1-(benzo[*d*]thiazol-2-yl)-5-(3-((4-methoxy-2,3,6-trimethylphenyl)sulfonyl)guanidino)-1-oxopentan-2-yl)carbamate

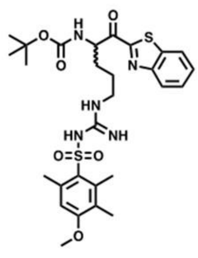

603.22 g/mol

HPLC

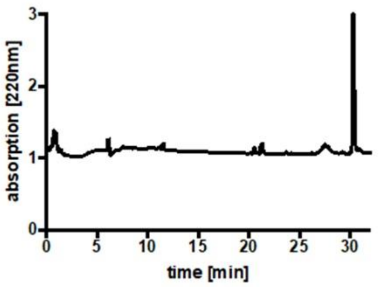

Retention time 30-31 min.
Purity (HPLC, 220 nm) > 90%.

NMR (300 MhZ, CD3OD)

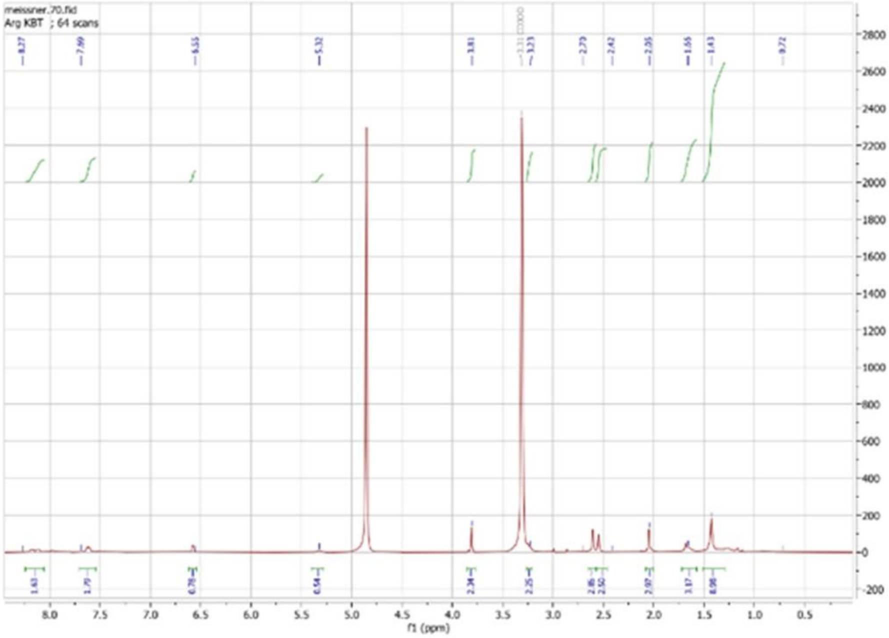

^{1}H NMR (300 MHz, CD3OD): δ=1.43 (s, 9 H) 1.66 (m, 4 H) 2.05 (s, 3 H) 2.55 (s, 3 H) 2.70 (s, 3 H) 3.23 (m, 2 H) 3.81 (s, 3 H) 5.32 (m, 1 H) 6.55 (s, 1 H) 7.69 (m, 2 H) 8.27 ppm (m, 2 H).

MS-ESI

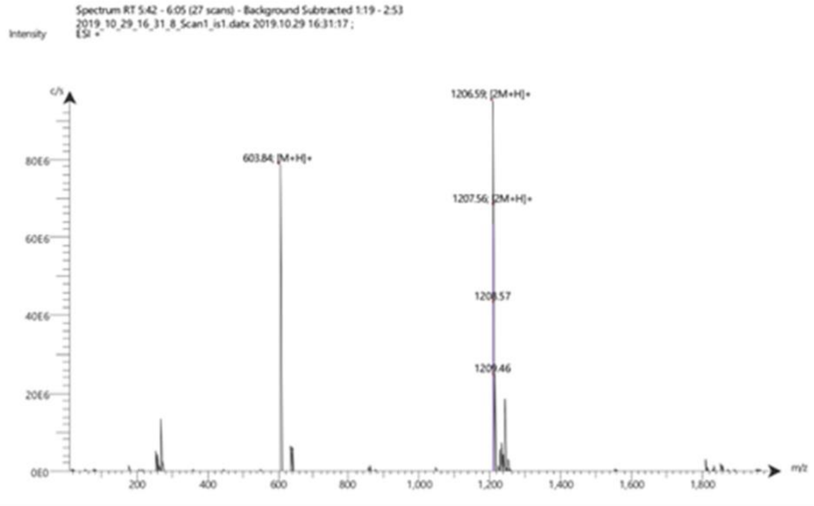

ESI (MS) *m/z*: calcd for $C_{28}H_{37}N_5O_6S_2$ $[M+H]^+$ 604.2, $[2M+H]^+$ 1207.4, found $[M+H]^+$ 603.9, $[2M+H]^+$ 1206.6

iii) **H_2N-Arg(Mtr) ketobenzothiazole (S3)**
(*N*-(*N*-(4-amino-5-(benzo[*d*]thiazol-2-yl)-5-oxopentyl)carbamimidoyl)-4-methoxy-2,3,6-trimethylbenzenesulfonamide

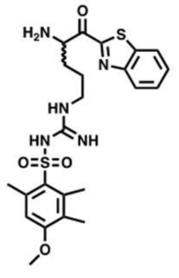

503.17 g/mol

HPLC

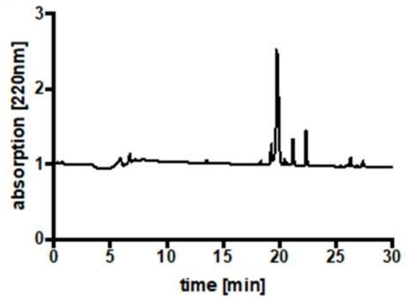

Retention time 19-20 min.
Purity (HPLC, 220 nm) > 80%

NMR (300 MhZ, CD3OD)

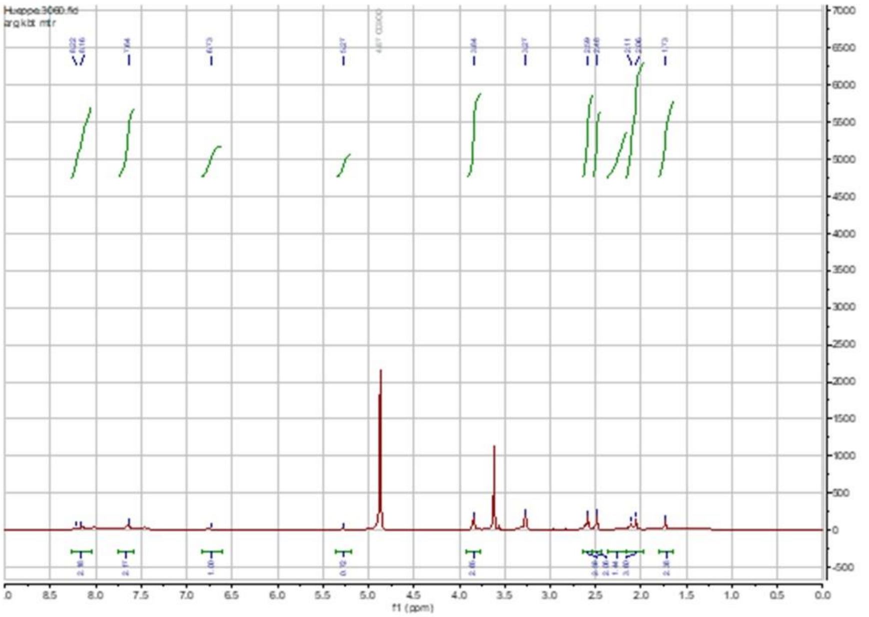

^{1}H NMR (300 MHz, CD3OD): δ = 1.73 (m, 2 H) 2.06–2.11 (m, 4 H) 2.24–2.36 (m, 1 H) 2.48 (s, 2 H) 2.59 (s, 3 H) 3.84 (s, 3 H) 5.27 (m, 1 H) 6.73 (s, 1 H) 7.64 (m, 2 H) 8.16–8.22 ppm (m, 2 H).

MS-ESI

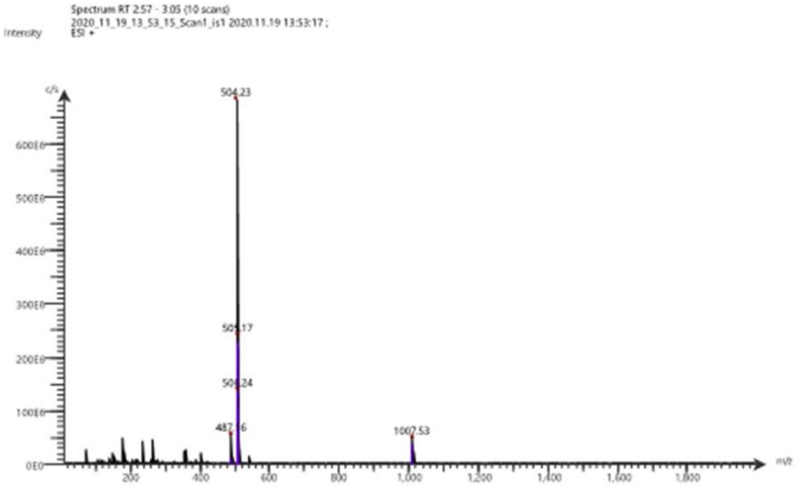

ESI (MS) *m/z*: calcd $[M+H]^+$ 504.2, $[2M+H]^+$ 1007.4 found $[M+H]^+$ 504.2, $[2M+H]^-$ 1007.5

iv) **Boc-Arg(Mtr) ketothiazole (S4)**

tert-Butyl-(5-(3-((4-methoxy-2,3,6-trimethylphenyl)sulfonyl)guanidino)-1-oxo-1-(thiazol-2-yl)pentan-2-yl)carbamate

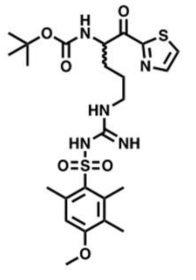

553.2 g/mol

NMR (300 MhZ, CD3Cl$_3$)

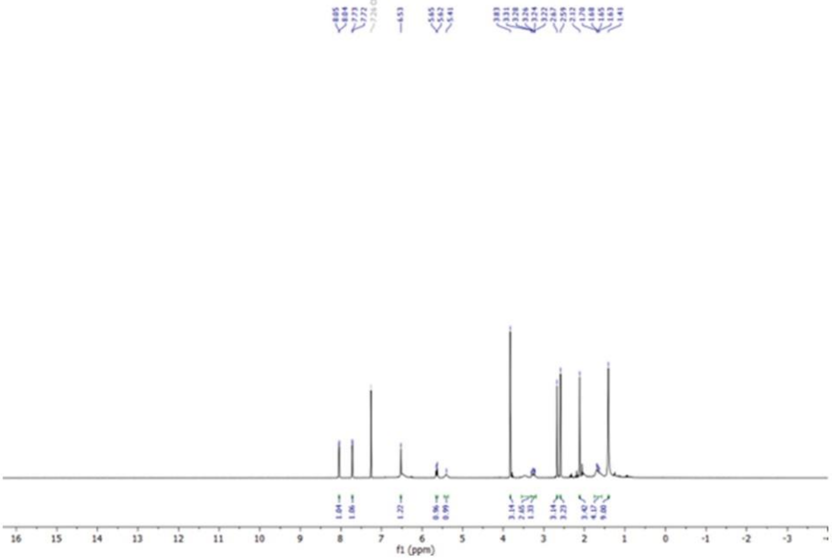

^{1}H NMR (300 MHz, $CDCl_3$): δ = 8.04 (d, 1 H), 7.72 (d, 1 H), 6.53 (s, 1 H), 5.64 (d, 1 H), 5.41 (s, 1 H), 3.83 (s, 3 H), 3.26 (m, 2 H), 2.67 (s, 3 H), 2.59 (s, 3 H), 2.12 (s, 3 H), 1.76 – 1.57 (m, 4 H), 1.41 (s, 9 H). ppm

LC-MS

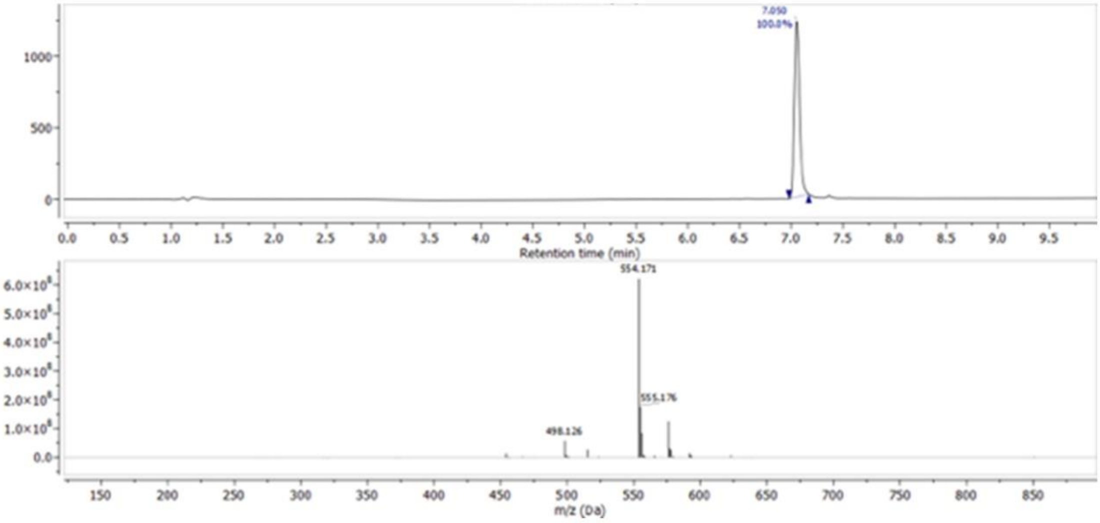

Retention time 7 min. Purity (254 nm) > 95 %

MS (ESI): *m/z*: calcd for $C_{24}H_{35}N_5O_6S_2$ $[M+H]^+$ 554.2, found $[M+H]^+$ 554.2

v) **H_2N-Arg(Mtr) ketothiazole (S5)**

N-(*N*-(4-amino-5-oxo-5-(thiazol-2-yl)pentyl)carbamimidoyl)-4-methoxy-2,3,6-trimethylbenzenesulfonamide

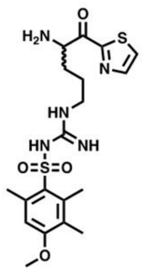

453.15 g/mol

LC-MS

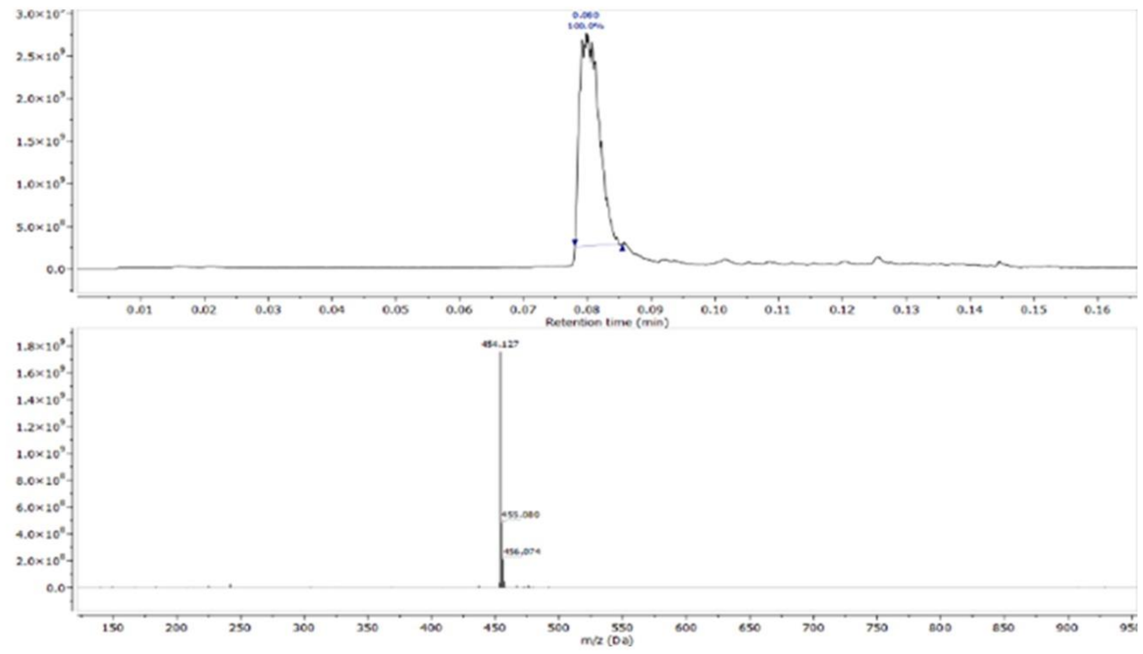

Retention time 0.08 min. Purity (254 nm) > 95 %

MS (ESI): *m/z*: calcd for $C_{20}H_{27}N_5O_4S_2$ $[M+H]^+$ 453.2, found $[M+H]^+$ 454.1.

vi) **Boc-Arg(Mtr) alcohol (S6)**

(*S*)-tert-Butyl-(1-hydroxy-5-(3-((4-methoxy-2,3,6-trimethylphenyl)sulfonyl)guanidino)pentan-2-yl)carbamate

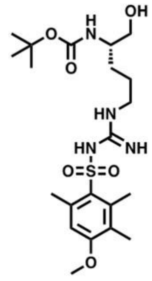

472.24 g/mol

NMR (300 MhZ, CD3Cl$_3$)

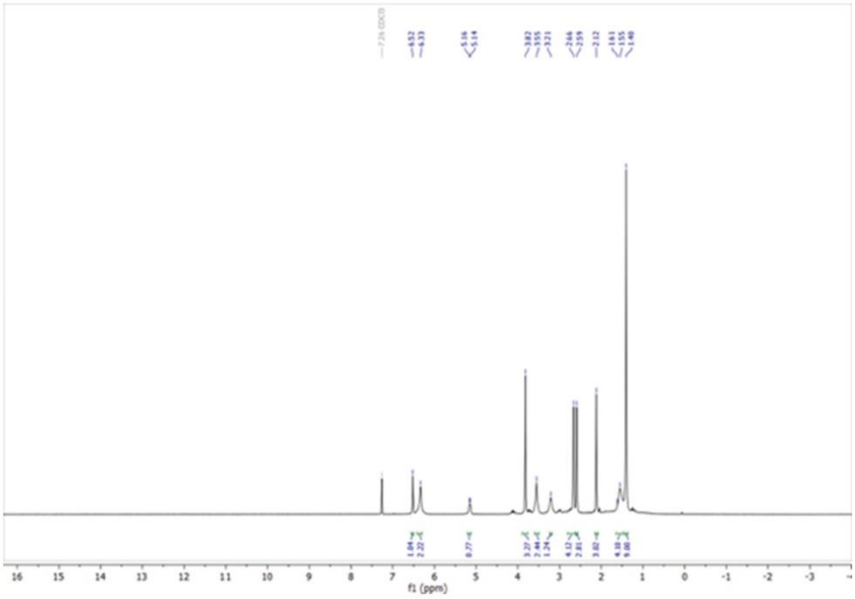

^{1}H NMR (300 MHz, $CDCl_3$) δ = 6.52 (s, 1 H), 6.33 (s, 2 H) 5.15 (d, 1 H), 3.82 (s, 3 H), 3.55 (s, 2 H), 3.21 (s, 1 H), 2.69–2.66 (m, 5 H), 2.59 (s, 3 H), 2.12 (s, 3 H), 1.55 (s, 4 H), 1.40 (s, 9 H) ppm

LC-MS

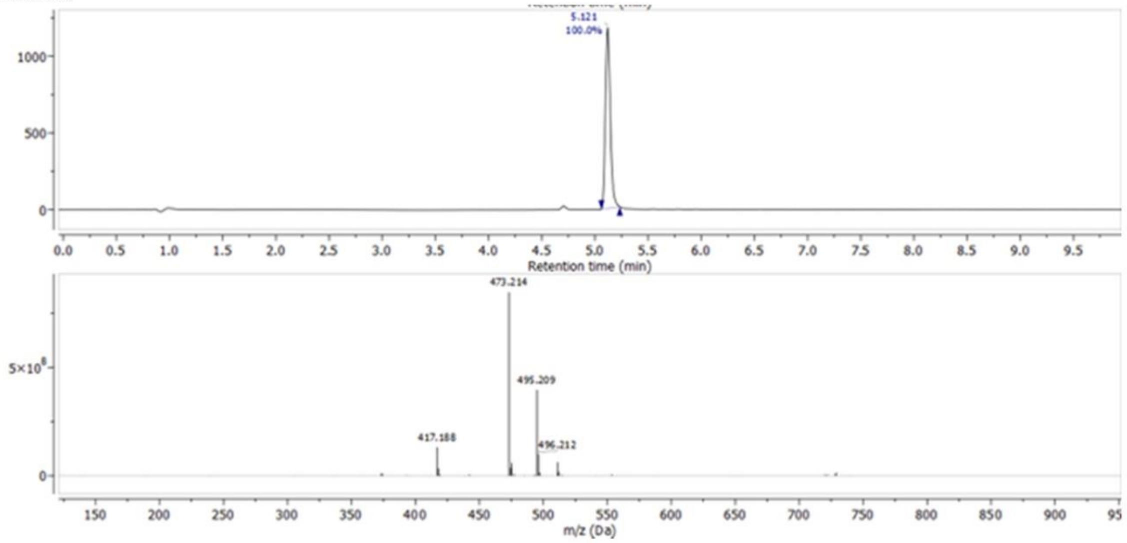

Retention time 5 min. Purity (254 nm) > 98 %

LC-MS *m/z*:calcd for $C_{21}H_{36}N_4O_6S$ $[M+H]^+$ 473.2, found $[M+H]^+$ 473.2.

vii) **H_2N-Arg(Mtr)-OH (S7)**
((*S*)-N-(N-(4-amino-5-hydroxypentyl)carbamimidoyl)-4-methoxy-2,3,6-trimethylbenzenesulfonamide)

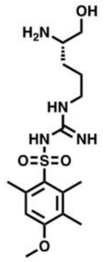

372.18 g/mol

HPLC

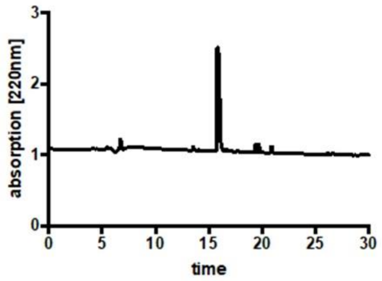

Retention time 16 min.
Purity (HPLC, 220 nm) > 90%.

MS-ESI

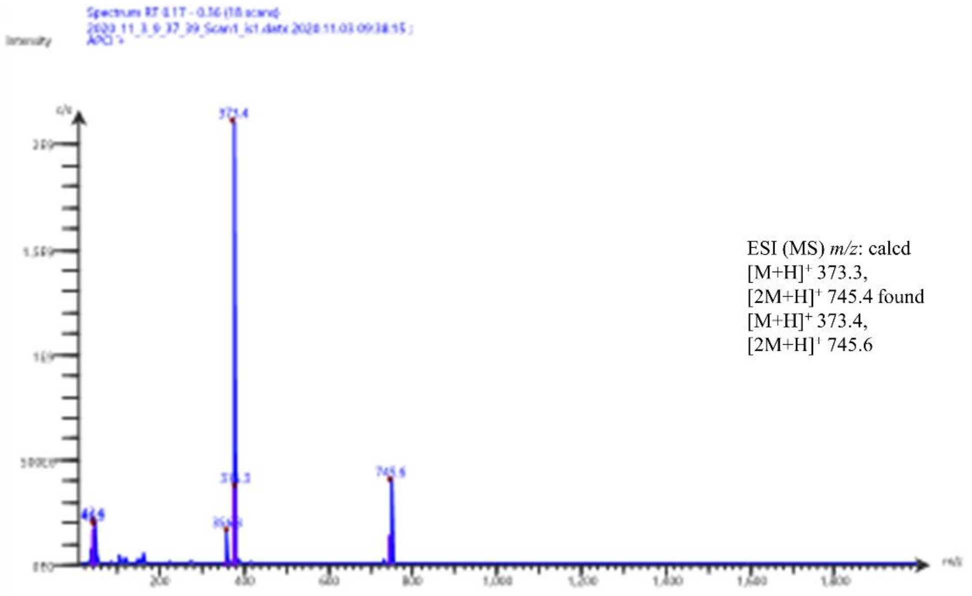

ESI (MS) *m/z*: calcd
$[M+H]^+$ 373.3,
$[2M+H]^+$ 745.4 found
$[M+H]^+$ 373.4,
$[2M+H]^+$ 745.6

Supplementary Figure 1 Analytical data (structure, HPLC, MS, NMR data) of precursors and serine traps

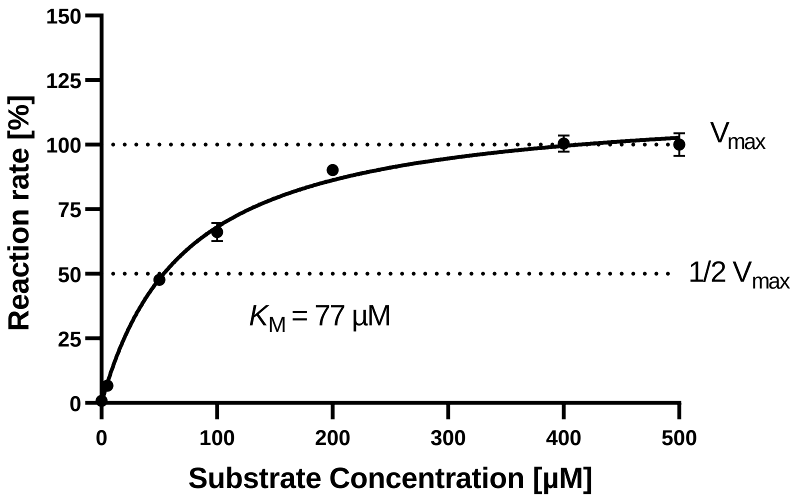

Supplementary Figure 2 Michaelis-Menten constant (*K*$_M$) of the fluorogenic reference substrate Boc-Gln-Ala-Arg-AMC for TMPRSS2. The data points were plotted using the Michaelis-Menten equation in GraphPad Prism prism version 8.4.2 (San Diego, California). Shown are the means ± SD of n=1 experiment performed in triplicates.

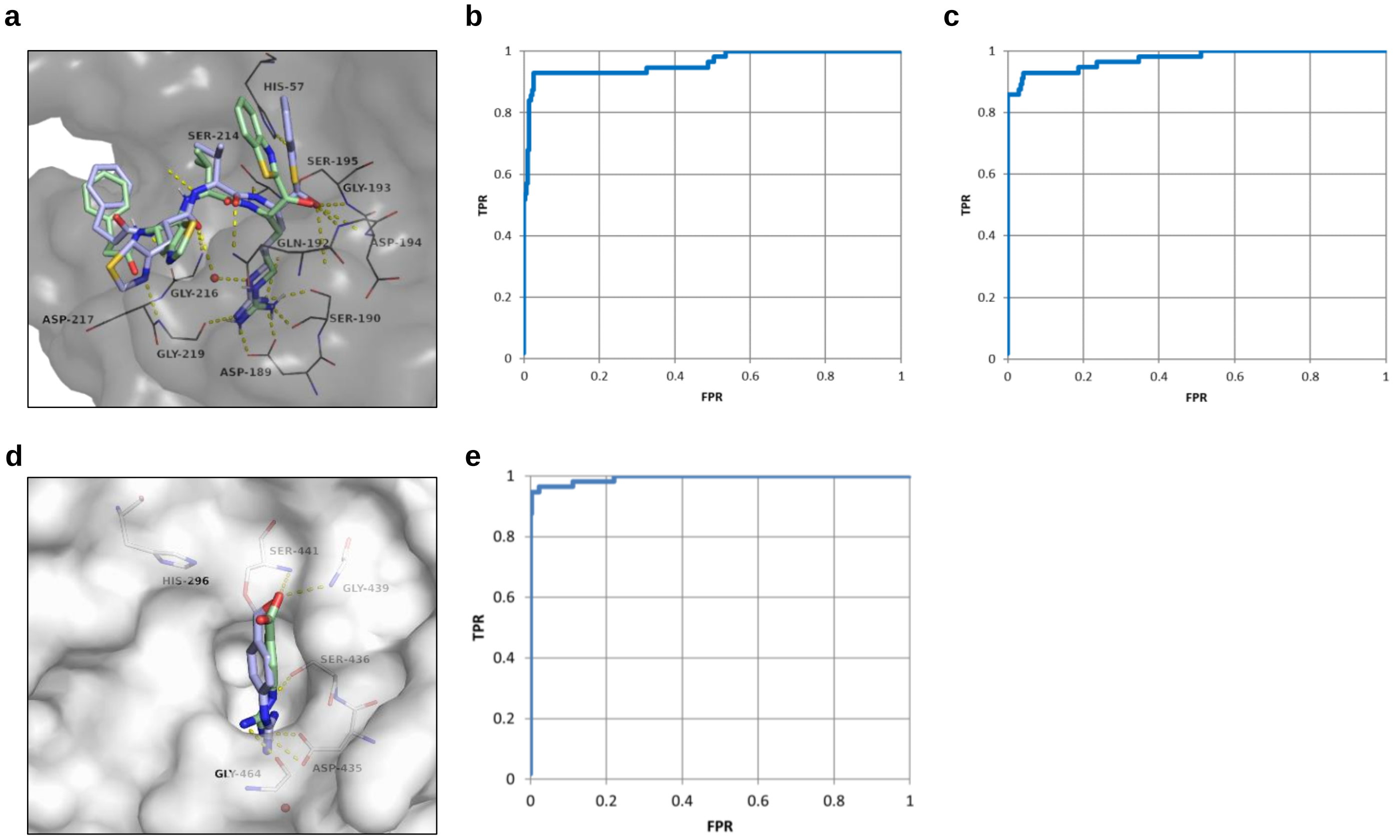

Supplementary Figure 3 Validation of molecular docking models. a) Re-docking of crystallographic ligand to the matriptase surrogate model (based on PDB-ID: 6N4T). Re-docked ligand: light green carbon atoms, crystallographic ligand: light blue carbon atoms, FlexX-score = -67.2 kJ/mol, RMSD = 1.8 Å. For clear view, only residues forming polar interactions (yellow dashed lines), and the catalytic residues Ser-195 and His-57 are depicted. b) ROC curve for binder vs. non-binder discrimination of molecular docking using matriptase surrogate model (PDB-ID: 6N4T, 56 binder and 314 non-binder, ROC AUC = 0.96). c) ROC curve for binder vs. non-binder discrimination of molecular docking using a TMPRSS2 homology model (56 binder and 314 non-binder, ROC AUC = 0.98). d) Redocking of crystallographic ligand (4-guanidinobenzoic acid) on TMPRSS2-nafamostat crystal structure (PDB-ID: 7MEQ, white carbon atoms and surface). Re-docked ligand: light green carbon atoms, crystallographic ligand: light blue carbon atoms, FlexX-score = -22.0 kJ/mol, RMSD = 1.6 Å. For clear view, only residues forming polar interactions with the ligand and catalytic Ser-441 and His-296 are depicted. e) ROC curve for binder vs. non-binder discrimination of molecular docking using TMPRSS2 crystal structure (PDB-ID: 7MEQ, 56 binder, 314 non-binder, ROC AUC = 0.99). At a FlexX-score cutoff of -34.2 kJ/mol one decoy is misclassified as a binder, while missing only two reported ligands. Noteworthy, covalent interactions are not considered in affinity prediction. FPR: false positive rate, TPR: true positive rate.

Cpd. 1

Structure	HPLC	MS-ESI
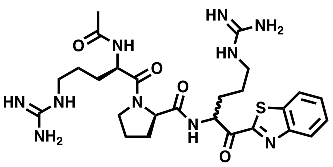	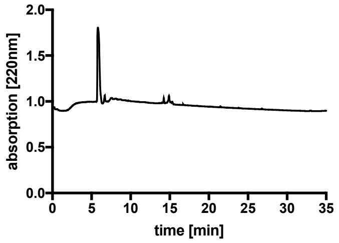	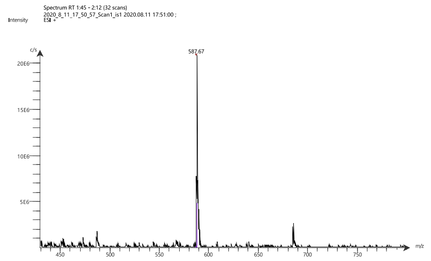
586.28 g/mol	Retention time 15.5 min. Purity (HPLC, 220 nm) > 90%.	ESI (MS) *m/z*: calcd. $[M+H]^+$ 587.3, $[2M+H]^+$ 1174.6 found $[M+H]^+$ 587.7.

Cpd. 2

Structure	HPLC	MS-ESI
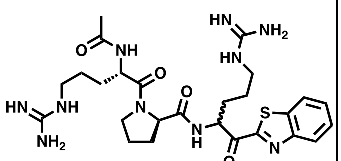	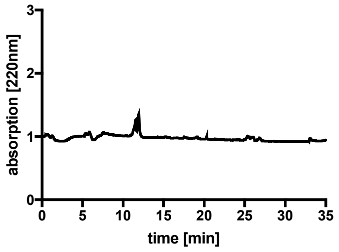	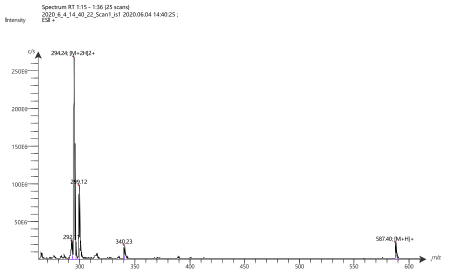
586.28 g/mol	Retention time 12.5 min. Purity (HPLC, 220 nm) > 90%.	ESI (MS) *m/z*: calcd. $[M+H]^+$ 587.3, $[M+2H]^{2+}$ 294.2 found $[M+H]^+$ 587.4, $[M+2H]^{2+}$ 294.2.

Cpd. 2*S*

Structure	HPLC	MS-ESI
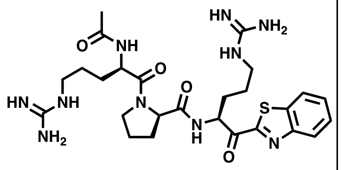	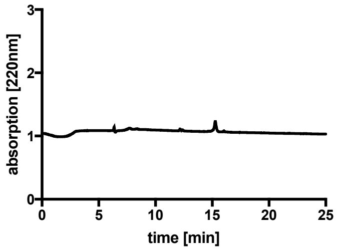	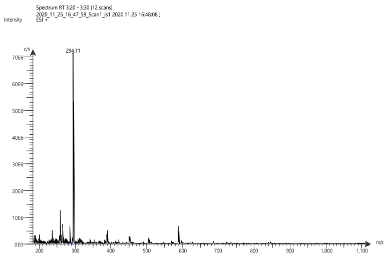
586.28 g/mol	Retention time 15.2 min. Purity (HPLC, 220 nm) > 90%.	ESI (MS) *m/z*: calcd. $[M+H]^+$ 587.3, $[M+2H]^{2+}$ 294.2 found $[M+H]^+$ 587.4, $[M+2H]^{2+}$ 294.1.

Cpd. 2*R*

Structure	HPLC	MS-ESI
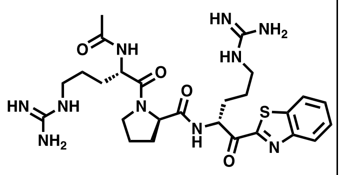	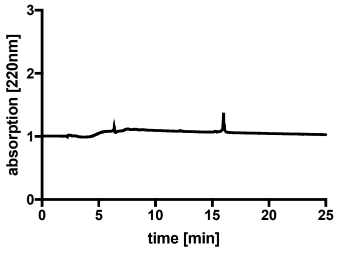	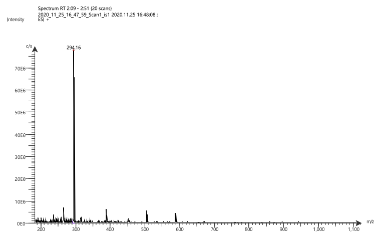
586.28 g/mol	Retention time 16.0 min. Purity (HPLC, 220 nm) > 90%.	ESI (MS) *m/z*: calcd. $[M+H]^+$ 587.3, $[M+2H]^{2+}$ 294.2 found $[M+H]^+$ 587.4, $[M+2H]^{2+}$ 294.1.

Cpd. 3

Structure	HPLC	MS-ESI
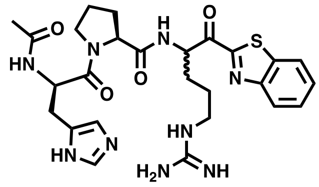	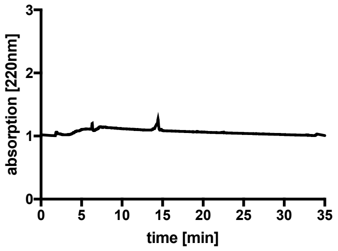	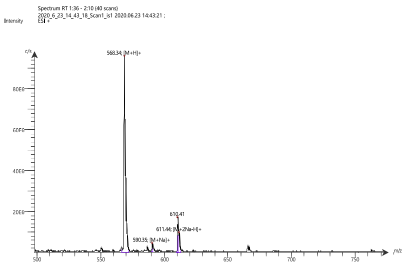
567.24 g/mol	Retention time 14.5 min. Purity (HPLC, 220 nm) > 90%.	ESI (MS) *m/z*: calcd. $[M+H]^+$ 568.2, $[M+Na]^+$ 590.2 found $[M+H]^+$ 568.3, $[M+Na]^+$ 590.3.

Cpd. 4

Structure	HPLC	MS-ESI
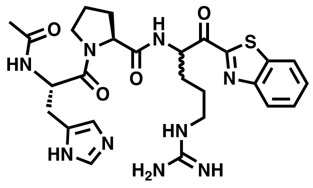	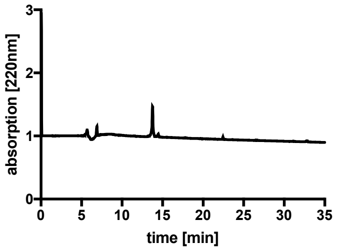	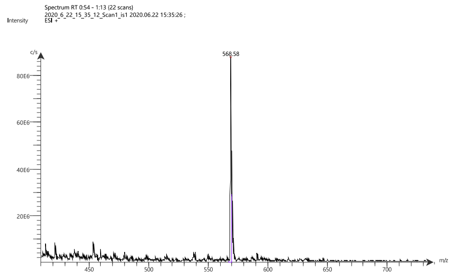
567.24 g/mol	Retention time 14.0 min. Purity (HPLC, 220 nm) > 90%.	ESI (MS) *m/z*: calcd. $[M+H]^+$ 568.2 found $[M+H]^+$ 568.6.

Cpd. 5

Structure	HPLC	MS-ESI
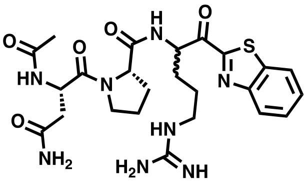	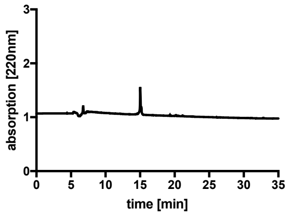	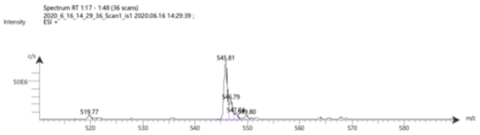
544.22 g/mol	Retention time 14.0 min. Purity (HPLC, 220 nm) > 90%.	ESI (MS) *m/z*: calcd. $[M+H]^+$ 545.2 found $[M+H]^+$ 545.8.

Cpd. 6

Structure	HPLC	MS-ESI
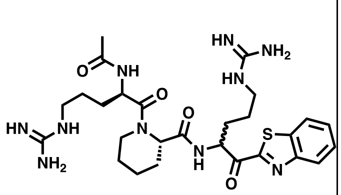	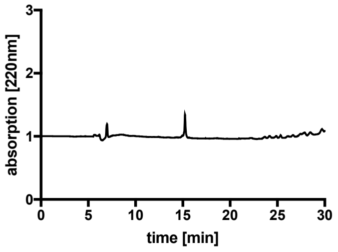	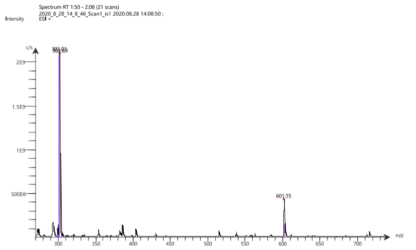
600.30 g/mol	Retention time 15.0 min. Purity (HPLC, 220 nm) > 90%.	ESI (MS) *m/z*: calcd. $[M+H]^+$ 601.3, $[M+2H]^{2+}$ 301.2, found $[M+H]^+$ 601.6 $[M+2H]^{2+}$ 301.7.

Cpd. 7

Structure	HPLC	MS-ESI
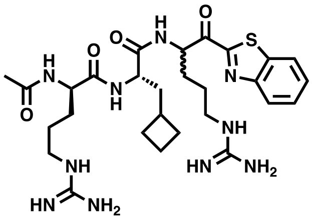<br>614.31 g/mol	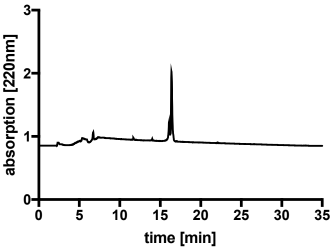<br>Retention time 16.0 min. Purity (HPLC, 220 nm) > 90%.	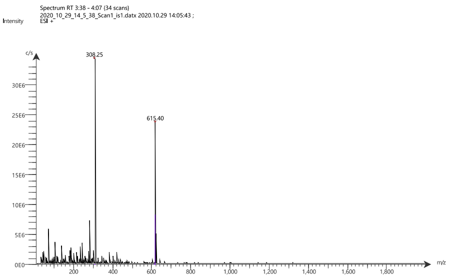<br>ESI (MS) *m/z*: calcd. $[M+H]^+$ 615.3, $[M+2H]^{2+}$ 308.2, found $[M+H]^+$ 615.4 $[M+2H]^{2+}$ 308.3.

Cpd. 7-2

Structure	HPLC	MS-ESI
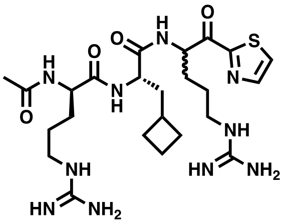<br>564.30 g/mol	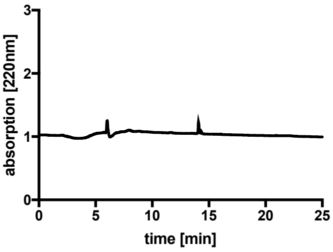<br>Retention time 14.0 min. Purity (HPLC, 220 nm) > 90%.	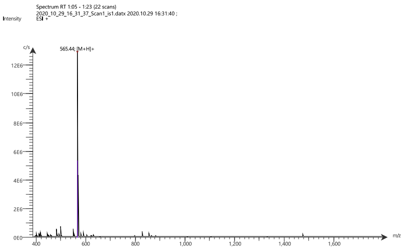<br>ESI (MS) *m/z*: calcd. $[M+H]^+$ 565.3 found $[M+H]^+$ 565.4.

Cpd. 7-3

Structure	HPLC	MS-ESI
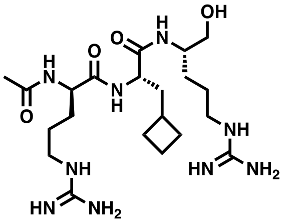<br>483.33 g/mol	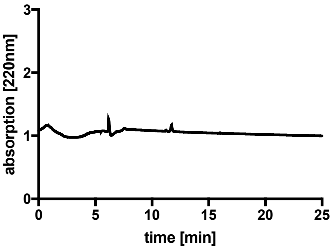<br>Retention time 12.0 min. Purity (HPLC, 220 nm) > 90%	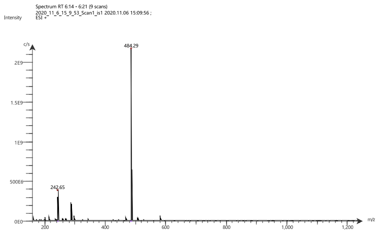<br>ESI (MS) *m/z*: calcd. $[M+H]^+$ 484.3, $[M+2H]^{2+}$ 242.7, found $[M+H]^+$ 484.3 $[M+2H]^{2+}$ 242.7.

Cpd. 8

Structure	HPLC	MS-ESI
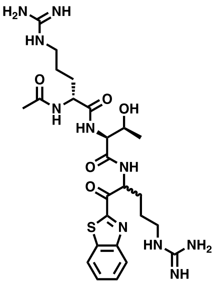<br>590.27 g/mol	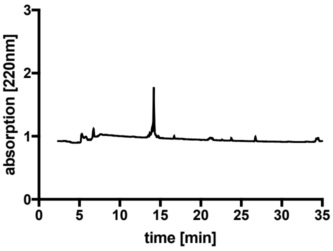<br>Retention time 14.0 min. Purity (HPLC, 220 nm) > 90%	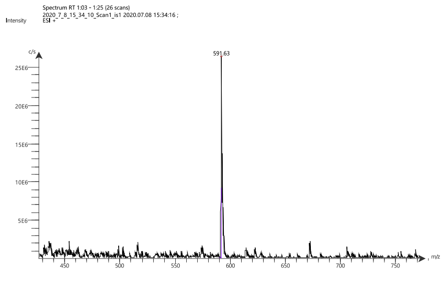<br>ESI (MS) *m/z*: calcd. $[M+H]^+$ 591.3 found $[M+H]^+$ 591.6.

Supplementary Figure 4 Analytical data (structure, HPLC, MS) of peptidomimetic inhibitors.

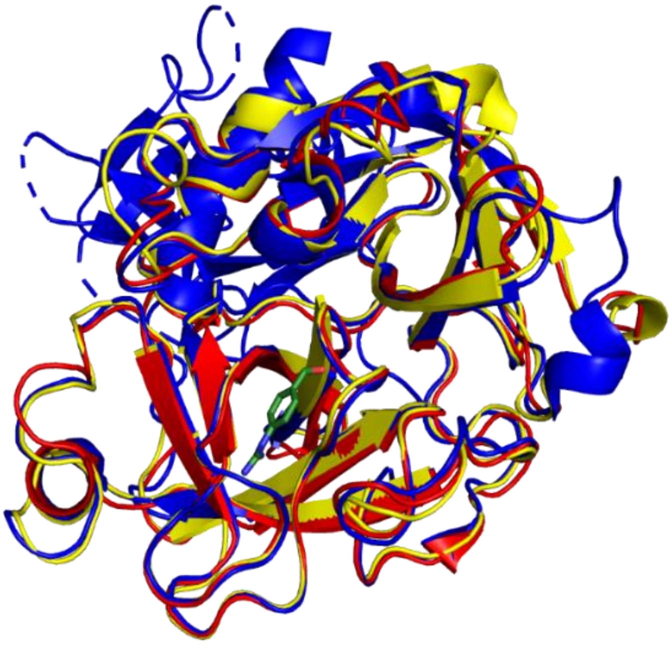

Supplementary Figure 5 Superposition of TMPRSS2 models and crystal structure. TMPRSS2 crystal structure (blue, PDB-ID: 7MEQ, crystallographic ligand in S1 shown with green carbon atoms for orientation), TMPRSS2 homology model (yellow, template hepsin, PDB-ID 1Z8G, C_α-RMSD compared to TMPRSS2 is 0.6 Å), matriptase (red, PDB-ID: 6N4T, C_α-RMSD compared to TMPRSS2 is 0.6 Å).

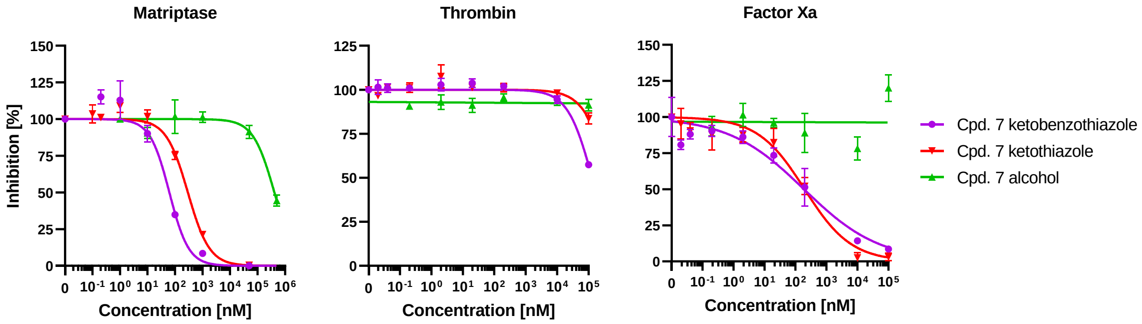

Supplementary Figure 6 Influence of serine trap on biological activity of compound 7 against matriptase, thrombin and factor Xa. Peptidomimetic inhibitors with a ketobenzothiazole, ketothiazole or alcohol serine trap residue were mixed with isolated enzymes. After 30 minutes, the fluorogenic reference substrate Boc-Gln-Ala-Arg-AMC was added to matriptase and the chromogenic substrates, D-Phe-Homopro-Arg-pNA or Bz-Ile-Glu-Gly-Arg-pNA, were added to thrombin or factor Xa, respectively. The velocity of substrate degradation was assessed by recording the fluorescence intensity at 460 nm or the absorbance at 405 nm within 2 h. Shown are the means ± SD n=1 experiment performed in triplicates.

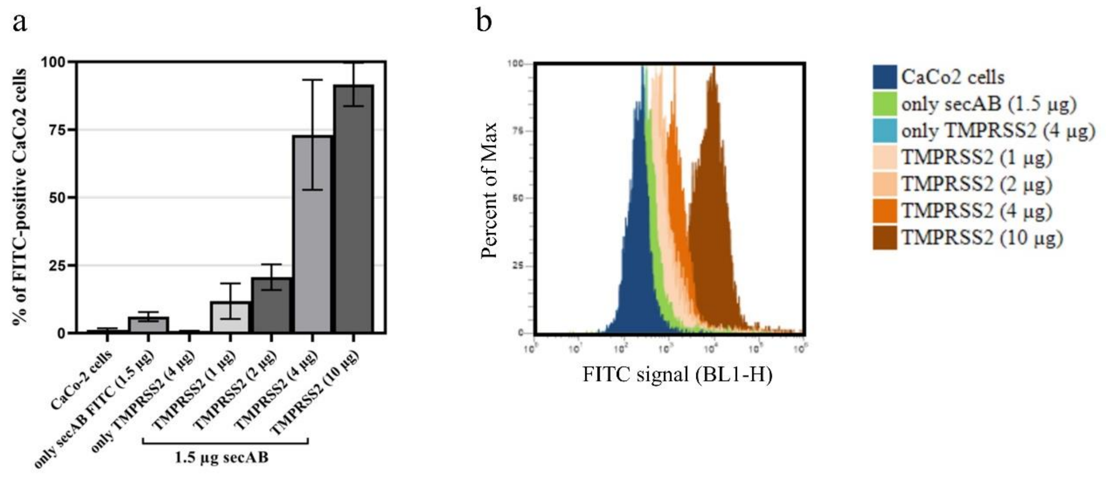

Supplementary Figure 7 The transmembrane serine protease TMPRSS2 is expressed on Caco-2 cells. Caco-2 cells were incubated with varying amounts of a TMPRSS2 antibody (ThermoFischer PA5-14264), followed with an anti-rabbit FITC-labeled secondary antibody (ThermoFischer A16024). Only secAB (secondary antibody) FITC and only TMPRSS2 were used as negative control. a) percentage of FITC-positive Caco-2 cells (n = 3, ± standard deviation). b) Overlay of the normalized FITC signal histograms for each group (one out of three histograms is exemplary shown for each group).

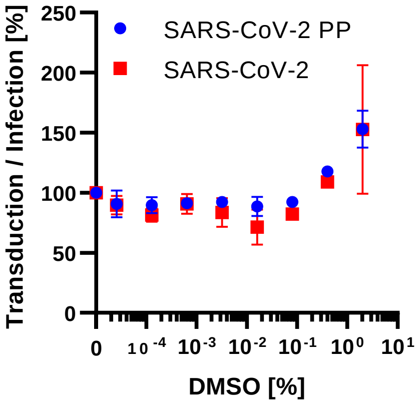

Supplementary Figure 8 Impact of DMSO on SARS-CoV-2 spike mediated entry and infection. DMSO was added to Caco-2 cells at concentrations corresponding to the maximum DMSO concentrations in transduction/infection experiments. After 1 h cells were either transduced with lentiviral SARS-CoV-2 pseudoparticles or infected with SARS-CoV-2 wildtype. Transduction/infection rates were assessed after 2 days by measuring luciferase activity in cell lysates or by ELISA, respectively. Shown are the mean ± SEM of n=2 experiments, performed in triplicates.

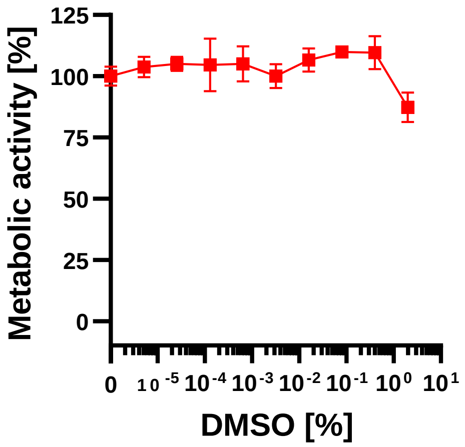

Supplementary Figure 9 Cytotoxicity of DMSO. DMSO concentrations corresponding to the maximum DMSO concentration applied were added to Caco2 cells. Cell viability was assessed 2 days post addition by measuring ATP content in cell lysates. Shown is the mean ± SD of n=1 experiment, performed in triplicates.

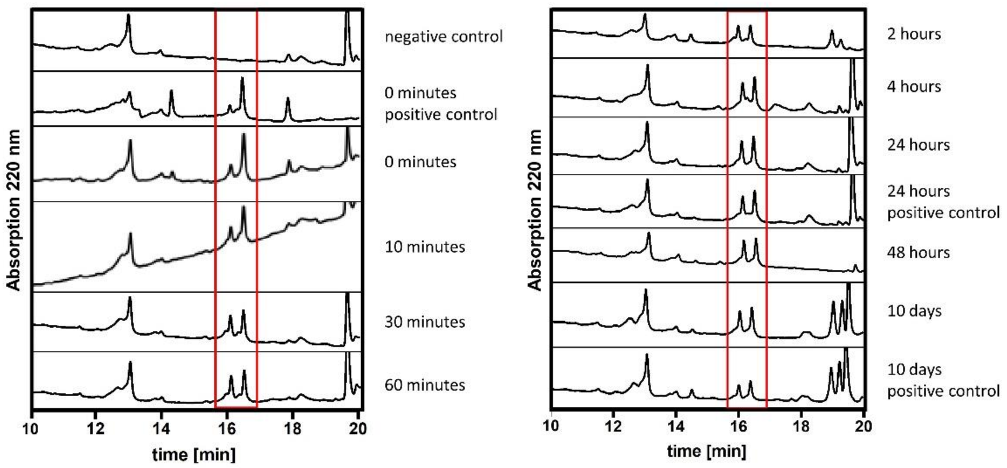

Supplementary Figure 10 UV absorbance profile (220 nm) of RP-HPLC chromatograms of 7 incubation with human serum. Two epimers can be observed D-Arg (Rt = 16 min) and L-Arg (Rt = 16:30 min). Negative control = 25% human serum in RPMI; positive control = inhibitor in pure RPMI medium.

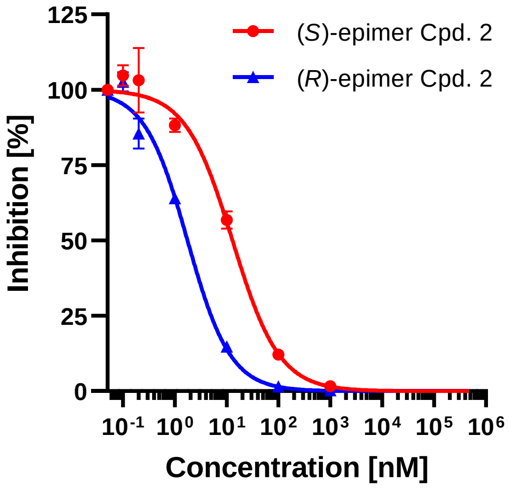

Compound	P1 Arg chirality	Matriptase K_i [nM]
**2*S*,*R***	*S*,*R*	3.3
**2*S***	*S*	6.2
**2*R***	*R*	0.9

Supplementary Figure 11 Influence of (*S*)- and (*R*)- epimers of compound 2 (ace-Arg-Pro-Arg-kbt) on inhibitory activity against matriptase. Shown is the mean ± SD of n=1 experiment, performed in triplicates.

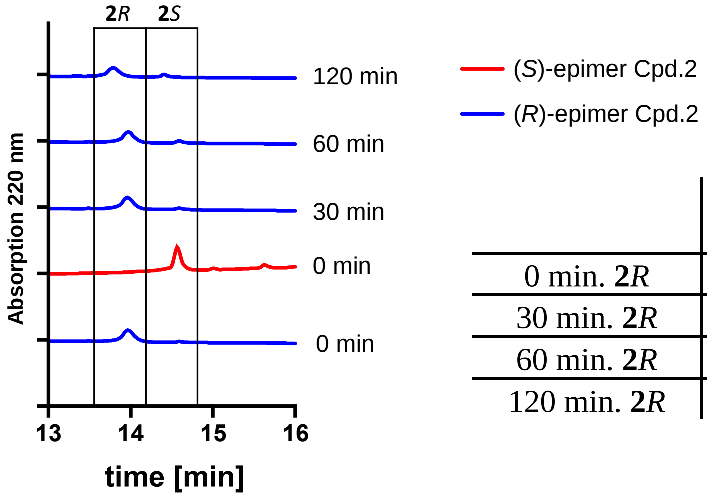

	**2**R:**2**S epimer
0 min. **2**R	96:4
30 min. **2**R	93:7
60 min. **2**R	89:11
120 min. **2**R	87:13

Supplementary Figure 12 Chromatogram of (*S*)- and (*R*)- epimer of compound 2. The chemical stability of compound 2*R* in TNC buffer at pH=8 with regard to racemization was analyzed at different time points 0 min, 30 min, 60 min and 120 min.

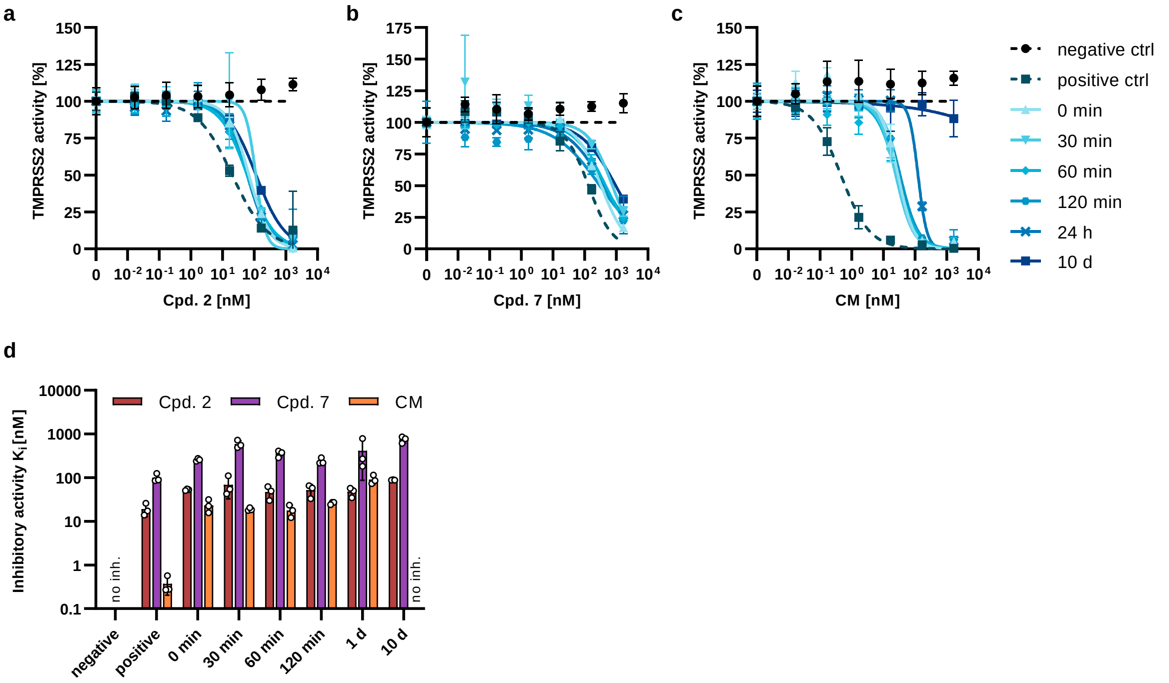

Supplementary Figure 13 Plasma stability of inhibitors. Compound 2 (a), compound 7 (b), or camostat mesylate (c) were incubated in human plasma for indicated timepoints. Samples were mixed with recombinant TMPRSS2, followed by the addition of the fluorogenic reference substrate BOC-Gln-Ala-Arg-AMC. Graph shows normalized fluorescence intensities after incubation for 2 h. negative ctrl: no inhibitor, positive ctrl: inhibitor in assay buffer. d) Inhibitory constants K_i as determined from (a-c). Shown are the means ± SD of n=1 experiment performed in triplicates. No inh.: no inhibition.

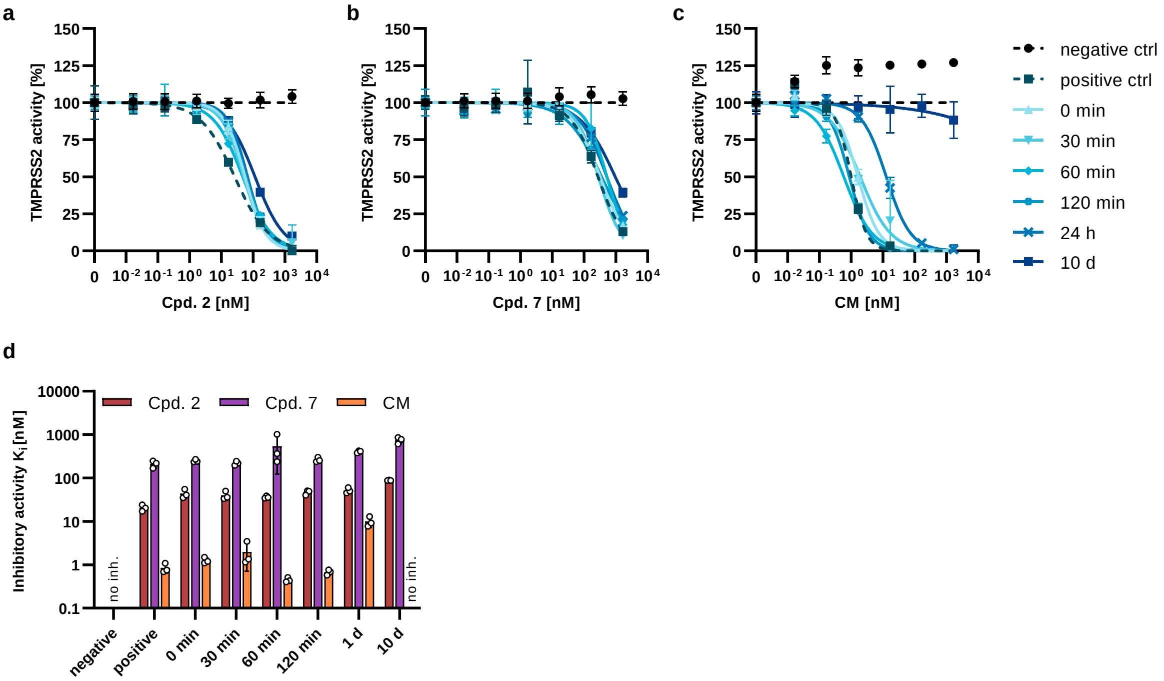

Supplementary Figure 14 Cell culture medium stability of inhibitors. Compound 2 (a), compound 7 (b), or camostat mesylate (c) were incubated in cell culture medium for indicated timepoints. Samples were mixed with recombinant TMPRSS2, followed by the addition of the fluorogenic reference substrate BOC-Gln-Ala-Arg-AMC. Graph shows normalized fluorescence intensities after incubation for 2 h. negative ctrl: no inhibitor, positive ctrl: inhibitor in assay buffer. d) Inhibitory constants K_i as determined from (a-c). Shown are the means ± SD of n=1 experiment performed in triplicates. No inh.: no inhibition.

Supplementary Table 1 FlexX-scores of tripeptidic substrate-analogue ligands for docking receptor validation. Scores are in kJ/mol, ace: N-terminal acetyl-cap, nme: C-terminal N-methylamide cap.

Ligand sequence	FlexX-score TMPRSS2 homology model	FlexX-score Matriptase surrogate
ace-D-Arg-Gly-Arg-nme	-48.8	-54.9
ace- D-Arg-Pro-Arg-nme	-53.8	-64.2
ace-Arg-Gly-Arg-nme	-51.1	-56.3
ace-Arg-Pro-Arg-nme	-48.0	-57.7

Supplementary Table 2 Docking results of P1-sidechain screening of peptidomimetic inhibitors with ace-Pro-X-aldehyde sequence for 7 potential basic amino acid-derivatives. X: position of altered amino acid, ace: N-terminal acetyl cap, mPG: meta-Phenylguanidine, pPG: para-Phenylguanidine, mBA: meta-Benzamidine, pBA: para-Benzamidine, Orn: Ornithin. Scores are in kJ/mol.

Ligand ace-Pro-X-Aldehyde	FlexX-score TMPRSS2 homology model	FlexX-score Matriptase surrogate
pBA	-51.2	-42.1
Arg	-45.1	-50.8
mPG	-40.1	-41.9
mBA	-33.9	-31.0
pPG	-28.3	-34.4
Lys	-22.9	-32.9
Orn	-19.9	-29.0

Supplementary Table 3 Excerpt of docking results of P2-sidechain screening of peptidomimetic inhibitors with ace-D-Arg-X-Arg-aldehyde sequence. A total of 369 molecules were docked. [a] Retrospective docking on the TMPRSS2 crystal structure (PDB-ID: 7MEQ) for peptide sequences selected for synthesis (scores of ketobenzothiazole (kbt)-coupled derivatives **1**, **6**, **7**, **8** in squared brackets). n.d. = not determined. Scores are in kJ/mol. AZc: Azetidine-2-carboxylic acid, Pip: Pipecolinic acid, Pgl: 3,4-Dichlorophenylglycine, Cyc: Cyclobutylalanine. [b]Predicted binding modes are shown in Figure 1a-c.

Ligand sequence ace-D-Arg-X-Arg-Aldehyde	FlexX-score (rank) TMPRSS2 homology model	FlexX-score (rank) Matriptase surrogate	FlexX-score TMPRSS2[a] [kbt; cpd x]
Arg	-57.4 (1)	-57.8 (59)	n.d.
Orn	-51.0 (2)	-59.7 (25)	n.d.
Pro[b]	-49.9 (8)	-60.5 (16)	-40.7 [-40.3; 1]
Pgl	-49.2 (9)	-58.8 (41)	n.d.
Phe	-48.4 (15)	-61.2 (9)	n.d.
Lys	-47.5 (23)	-58.6 (45)	n.d.
Thr	-46.9 (29)	-62.2 (4)	-32.7 [-42.5; 8]
Azc	-46.9 (39)	-60.1 (22)	n.d.
Val	-45.9 (54)	-62.1 (6)	n.d.
Pip	-44.1 (101)	-55.9 (110)	-39.6 [-33.2; 6]
Leu	-43.5 (127)	-56.6 (96)	n.d.
Ile	-41.9 (183)	-61.5 (8)	n.d.
Cyc	-39.8 (269)	-52.9 (227)	-32.1 [-35.4; 7]

Supplementary Table 4 Excerpt of docking results of P3-sidechain screening of peptidomimetic inhibitors with ace-X-Pro/Gly-Arg-aldehyde sequence. For both X-Pro-Arg and X-Gly-Arg sequences 388 molecules were docked and ranked separately. [a] Retrospective docking on the TMPRSS2 crystal structure (PDB-ID: 7MEQ) for peptide sequences selected for synthesis (scores of ketobenzothiazole (kbt)-coupled derivatives **1-5** in squared brackets). Scores are in kJ/mol. n.d. = not determined.

Ligand sequence ace-X-Y-Arg-Aldehyde	FlexX-score (rank) TMPRSS2 homology model		FlexX-score (rank) Matriptase surrogate		FlexX-score TMPRSS2[a] [kbt; cpd x]
X	Y = Pro	Y = Gly	Y = Pro	Y = Gly	Y = Pro
Arg	-52.0 (1)	-48.3 (3)	-55.1 (298)	-56.4 (28)	-34.3 [-44.0; 2]
D-Arg	-44.2 (135)	-45.5 (10)	-64.9 (1)	-56.4 (27)	-40.7 [-40.3; 1]
His	-39.7 (348)	-40.5 (144)	-54.5 (364)	-54.3 (90)	-35.4 [-41.4; 4]
D-His	-49.3 (6)	-41.8 (92)	-62.2 (16)	-57.4 (6)	-34.3 [-41.9; 3]
Trp	-45.1 (97)	-44.2 (20)	-63.5 (7)	-54,7 (71)	n.d.
D-Trp	-45.1 (104)	-42.8 (50)	-59.7 (70)	53.5 (122)	n.d.
Asn	-40.5 (329)	-40.2 (160)	-52.9 (364)	-56.5 (23)	-36.1 [-42.3; 5]
D-Asn	-46.7 (47)	-42.1 (78)	-57.1 (190)	-51.8 (201)	n.d.
Met	-42.3 (253)	-38.4 (261)	-57.8 (158)	-47.9 (369)	n.d.
D-Met	-38.7 (370)	-35.8 (368)	-59.4 (77)	-50.1 (281)	n.d.
Glu	-39.1 (362)	-40.8 (127)	-54.3 (323)	-50.9 (241)	n.d.
D-Glu	-40.6 (325)	-38.6 (252)	-50.6 (384)	-51.9 (193)	n.d.

Supplementary Table 5 Influence of serine trap on biological activity of compound 7 against matriptase, thrombin and factor Xa. The ketobenzothiazole (cpd. 7) serine trap moiety was truncated to ketothiazole and further reduced to the alcohol for complete abolishment of electrophilicity. n.i. = no inhibition within concentration range.

	K_i [nM]		
Compound	Matriptase	Thrombin	Factor Xa
Cpd. 7 ketobenzothiazole	30	n.i.	94.1
Cpd. 7 ketothiazole	141.2	n.i.	98.7
Cpd. 7 alcohol	n.i.	n.i.	n.i.

Supplementary Table 6 IC_{50} values of peptidomimetic TMPRSS2 inhibitors, camostat mesylate (CM) and FOY-251 measured on Caco-2 or TMPRSS2 expressing HEK 293T cells. n.d.: not determined.

Compound	IC_{50} Caco2 [nM]	IC_{50} HEK293T TMPRSS2 [nM]
2	32	3.5
4	45.5	13.3
5	12.7	2.2
7	234.2	27.9
CM	42.2	1.1
FOY-251	377.2	n.d.

Supplementary Table 7 IC_{50} values of peptidomimetic TMPRSS2 inhibitors and camostat mesylate (CM) against SARS-CoV-2 spike -pseudotyped lentivirus. n.d.: not determined.

	IC_{50} [nM]				
Compound	Wuhan-Hu-1	Alpha	Beta	Delta	Omicron BA.1
1	1,201	n.d.	n.d.	n.d.	n.d.
2	1,617	390.5	433.9	627.0	431.0
3	3,243	n.d.	n.d.	n.d.	n.d.
4	1,322	485.7	141.2	610.2	338.8
5	467.2	260.7	153.3	286.1	196.3
6	12,085	n.d.	n.d.	n.d.	n.d.
7	2,068	1,597	920.8	2,367	1,701
8	5,604	n.d.	n.d.	n.d.	n.d.
CM	747.5	98.73	156.4	85.48	77.62

Supplementary Table 8 IC_{50} values of peptidomimetic TMPRSS2 inhibitors and camostat mesylate (CM) against SARS-CoV-2 Wuhan-Hu-1 and variants of concern. n.d.: not determined.

	IC_{50} [nM]			
Compound	Wuhan-Hu-1	Wuhan-Hu-1 D614G	Alpha	Beta
1	18,889	n.d.	n.d.	n.d.
2	4,567	17,126	~20,000	6,880
3	12,569	n.d.	n.d.	n.d.
4	5,667	15,405	~20,000	6,985
5	4,654	11,867	6,782	6,260
6	>100,000	n.d.	n.d.	n.d.
7	20,427	51,170	21,649	24,011
8	24,460	n.d.	n.d.	n.d.
CM	3,625	26,672	16,407	9,307
