# Supplementary material for: Peptidomimetic inhibitors of TMPRSS2 block SARS-CoV-2 infection in cell culture
Source: Commun Biol. 2022 Jul 8;5:681. doi: 10.1038/s42003-022-03613-4 (PMC9270327; doi:10.1038/s42003-022-03613-4)
Supplement: Supplementary file 5 — Reporting Summary [file 42003_2022_3613_MOESM5_ESM.pdf]

nature research

Corresponding author(s): Volker Mailänder, Jan Münch

Last updated by author(s): May 16, 2022

Reporting Summary

Nature Research wishes to improve the reproducibility of the work that we publish. This form provides structure for consistency and transparency in reporting. For further information on Nature Research policies, see our Editorial Policies and the Editorial Policy Checklist.

Statistics

For all statistical analyses, confirm that the following items are present in the figure legend, table legend, main text, or Methods section.

n/a	Confirmed	
☐	☒	The exact sample size (n) for each experimental group/condition, given as a discrete number and unit of measurement
☐	☒	A statement on whether measurements were taken from distinct samples or whether the same sample was measured repeatedly
☒	☐	The statistical test(s) used AND whether they are one- or two-sided<br>*Only common tests should be described solely by name; describe more complex techniques in the Methods section.*
☒	☐	A description of all covariates tested
☐	☒	A description of any assumptions or corrections, such as tests of normality and adjustment for multiple comparisons
☐	☒	A full description of the statistical parameters including central tendency (e.g. means) or other basic estimates (e.g. regression coefficient) AND variation (e.g. standard deviation) or associated estimates of uncertainty (e.g. confidence intervals)
☒	☐	For null hypothesis testing, the test statistic (e.g. F, t, r) with confidence intervals, effect sizes, degrees of freedom and P value noted<br>*Give P values as exact values whenever suitable.*
☒	☐	For Bayesian analysis, information on the choice of priors and Markov chain Monte Carlo settings
☒	☐	For hierarchical and complex designs, identification of the appropriate level for tests and full reporting of outcomes
☒	☐	Estimates of effect sizes (e.g. Cohen's d, Pearson's r), indicating how they were calculated

Our web collection on statistics for biologists contains articles on many of the points above.

Software and code

Policy information about availability of computer code

Data collection	LeadIT-2.3.2, CycloPs 8.5.9, Swiss-Model (free for academic use), ZINK database (free for academic use), MOE2019
Data analysis	GraphPad Prism version 8.4.2, GraphPad Prism version 7.0a

For manuscripts utilizing custom algorithms or software that are central to the research but not yet described in published literature, software must be made available to editors and reviewers. We strongly encourage code deposition in a community repository (e.g. GitHub). See the Nature Research guidelines for submitting code & software for further information.

Data

Policy information about availability of data

All manuscripts must include a data availability statement. This statement should provide the following information, where applicable:
- Accession codes, unique identifiers, or web links for publicly available datasets
- A list of figures that have associated raw data
- A description of any restrictions on data availability

Crystal structures were obtained from Protein Data Bank with accession codes PDB ID: 1Z8G, 6N4T and from SWISS-MODEL repository (https://swissmodel.expasy.org/repository/uniprot/P05981?template=1z8g). Source data are provided with this paper.

Field-specific reporting

Please select the one below that is the best fit for your research. If you are not sure, read the appropriate sections before making your selection.

☒ Life sciences ☐ Behavioural & social sciences ☐ Ecological, evolutionary & environmental sciences

For a reference copy of the document with all sections, see nature.com/documents/nr-reporting-summary-flat.pdf

Life sciences study design

All studies must disclose on these points even when the disclosure is negative.

Sample size	Sample sizes were not calculated. Screening assays were performed in one or three independent experiments due to limited screening material. Sample sizes were determined based on established protocols.
Data exclusions	For inhibitor assays, low and inactive concentrations were omitted for clarity of graphical presentation. This did not affect the reported activities.
Replication	The number of replicates is indicated in the respective figure legends.
Randomization	Randomization is not applicable to this study.
Blinding	Blinding is not applicable to this study.

Reporting for specific materials, systems and methods

We require information from authors about some types of materials, experimental systems and methods used in many studies. Here, indicate whether each material, system or method listed is relevant to your study. If you are not sure if a list item applies to your research, read the appropriate section before selecting a response.

Materials & experimental systems

n/a	Involved in the study	
☐	☒	Antibodies
☐	☒	Eukaryotic cell lines
☒	☐	Palaeontology and archaeology
☒	☐	Animals and other organisms
☒	☐	Human research participants
☒	☐	Clinical data
☒	☐	Dual use research of concern

Methods

n/a	Involved in the study	
☒	☐	ChIP-seq
☐	☒	Flow cytometry
☒	☐	MRI-based neuroimaging

Antibodies

Antibodies used	TMPRSS2 antibody (rabbit anti-human IgG from ThermoFisher Scientific, PA5-14264)<br>FITC-labeled secondary donkey anti-rabbit IgG (ThermoFisher, A16024)
Validation	TMPRSS2 polyclonal antibody- Rabbit IgG, antibody validation by the manufacturer: WB with 293 and NCI-H460 cell lysates; IHC on testis tissue; FC on 293 cells, publications e.g. Zhou et al. https://doi.org/10.1038/s41591-020-0912-6, Encabo et al. https://doi.org/10.1016/j.stemcr.2021.02.001.<br>FITC-labeled secondary donkey anti-rabbit IgG validation by the manufacturer: Purification by affinity chromatography; Confirmation of sensitivity by ELISA; Confirmation of specificity by isoelectric focusing (IEF)

Eukaryotic cell lines

Policy information about cell lines

Cell line source(s)	HEK293T cells were obtained from ATCC.<br>Vero E6 cells were obtained from National Institute for Biological Standards and Control.<br>Caco-2 cells were obtained from ATCC (ATCC® HTB-37™) and DSMZ (ACC 169).
Authentication	HEK293T cells were authenticated by Multiplex human Cell line Authentication Test (MCA)(SNP-Profiling).<br>The DSMZ and ATCC company assure largerly authenticated cell lines based on a comprehensive quality control program. This quality control program evaluates growth characteristics and optimal culture conditions.

Mycoplasma contamination	Major attention is focused on testing microbial contaminations caused by bacteria (notably mycoplasmas), fungi and yeasts. Additionally, all primate cell lines are screened for certain human pathogenic viruses.
Commonly misidentified lines (See ICLAC register)	None.

Flow Cytometry

Plots

Confirm that:

☒ The axis labels state the marker and fluorochrome used (e.g. CD4-FITC).

☒ The axis scales are clearly visible. Include numbers along axes only for bottom left plot of group (a 'group' is an analysis of identical markers).

☒ All plots are contour plots with outliers or pseudocolor plots.

☒ A numerical value for number of cells or percentage (with statistics) is provided.

Methodology

Sample preparation	Caco-2 cells were first incubated with different amounts of a TMPRSS2 antibody (ThermoFisher Scientific, PA5-14264, rabbit anti-human) followed by the detection of bound TMPRSS2 antibodies on the cell surface via an anti-rabbit FITC-labeled secondary antibody. Only secAB (secondary antibody) FITC and only TMPRSS2 were used as negative control groups.
Instrument	Attune™ NxT cytometer (ThermoFisher)
Software	Attune™ NxT software (ThermoFisher)
Cell population abundance	For our purpose, post-sorting of cell populations was not necessary because only the CaCo-2 cell line was used.
Gating strategy	Caco-2 cells were selected by the FSC/SSC plot, thereby excluding cell debris. From this dot plot gating of Caco-2 cells, a histogram plot of the BL1-H emission filter signal was generated. The signal of untreated Caco-2 cells (autofluorescence) was gated to one percent, whereby all other samples refer to the percentage of events within this gate.

☐ Tick this box to confirm that a figure exemplifying the gating strategy is provided in the Supplementary Information.
